# Study protocol design and evaluation of a hospital-based multi-professional educational intervention: Person-Centred Psychosis Care (PCPC)

**DOI:** 10.1186/s12888-018-1852-2

**Published:** 2018-08-30

**Authors:** Anneli Goulding, Katarina Allerby, Lilas Ali, Andreas Gremyr, Margda Waern

**Affiliations:** 1 Psychosis Clinic, Sahlgrenska University Hospital, Mölndalsvägen 31 hus V, 431 80 Mölndal, Gothenburg, Sweden; 2 Psychology Department, University of Gothenburg, Gothenburg, Sweden; 3 Section of Psychiatry and Neurochemistry, Institute of Neuroscience and Physiology, Sahlgrenska Academy, University of Gothenburg, SU Sahlgrenska, 413 45 Gothenburg, Sweden; 4 Institute of Health Care Sciences, Centre for Person-Centred Care, Sahlgrenska Academy, University of Gothenburg, Box 457, 405 30 Gothenburg, Sweden

**Keywords:** Person-centred psychosis care, Psychiatry, Schizophrenia, Education, Intervention, Implementation, Empowerment, Consumer satisfaction, Involuntary treatment

## Abstract

**Background:**

While patient involvement in mental health care is repeatedly stressed in policy documents, there are actually few studies that evaluate person-centred care interventions within psychiatric services. We present here the design and planned evaluation of an educational intervention for inpatient staff involved in the care of persons with schizophrenia and similar psychoses.

**Methods/design:**

The care intervention will be assessed using a non-randomised trial with a before and after approach. The intervention involves an educational and experimental learning phase for hospital staff, followed by an implementation phase. The intervention is multi-professional; psychiatrists, psychiatric nurses, psychiatric carers, social workers, occupational therapists, and a medical secretary will be engaged in a participatory approach where they practice how to create a partnership and explore recovery-related goals together with patients. Patient-related outcomes include empowerment and satisfaction with care. Ward-level outcomes include daily ward burden, length of inpatient stay, and number of days with involuntary care. In addition, qualitative methods will be applied to capture patient, next-of-kin, and staff perspectives.

**Discussion:**

The care intervention is expected to contribute to the improvement of inpatient care for persons with severe and complex mental health issues.

**Trial registration:**

The trial was retrospectively registered at ClinicalTrials.gov June 9, 2017, identifier: NCT03182283.

## Background

Schizophrenia affects approximately 1% of the population and, because it is often associated with early onset and long term, severe disability, it has a major impact on Disability-Adjusted Life Years worldwide [1]. For the families of persons with schizophrenia, the burden of care is great [1–3].

The treatment of schizophrenia rests on three cornerstones: 1) pharmacological treatment with the lowest effective dose of anti-psychotic medication, 2) psychological and psychosocial treatments, and 3) social support [2]. Treatment with anti-psychotics is often a prerequisite for symptom control. While patients and their relatives alike recognize this, they experience that too much focus on pharmacological treatment can hamper the initiation of other treatment elements, and lead to feelings of powerlessness and resignation [4]. Further, care outcomes of importance for the patient may differ from those commonly utilized in traditional medical treatment settings [5].

A systematic review highlighted the need for research on patient involvement and the organization of care for persons with schizophrenia [6]. The report stressed the importance of increased involvement in the care process on the part of service users and their families. This theme has been reiterated in policy statements from a number of Western countries during the past decade [7], yet a large gap exists between these recommendations and real world practices [8].

### Person-centred care

The concept of person-centred care has a long history. Introducing the term in the 1940s, Carl Rogers [9] highlighted three important principles: every person possesses considerable qualities, can make use of available resources, and can find a way to remedy difficulties. Many definitions of person-centred care have been proposed since then, and there is to date no clear consensus definition [10]. A recent overview of reviews on the person-centred care concept [11] identified six components of person-centred care: establishing a therapeutic relationship, sharing power and responsibility, getting to know the patient as a person, working to empower the person, creating and working with trust and respect, and communicating understandable and accurate information. We define person-centred care according to these components throughout the present paper.

Person-centred care outcome studies have been conducted in a variety of patient populations and settings [12–14], but relatively few research studies target inpatient psychiatric services. Out of 34 publications identified in a recent review relating to person-centred care in psychiatric inpatient settings [15], only 16 were described as involving original research. Most of these did not evaluate person-centred care interventions but instead described nurses’ caring approaches, the development of self-report measures, and evaluations of specific information tools.

Three studies that evaluated person-centred care interventions were included in the review [15], one was described as an original research paper, the other two as practice development papers although they reported on intervention outcomes. The first study [16], reported on a series of person-centred care interventions implemented at 58 different psychiatric emergency and inpatient psychiatric units. The person-centred care interventions were associated with a reduction of seclusion and restraint use. The second study [17] also reported on person-centred care interventions and these were associated with culture change, a restraint-free environment, and less medication. The third study [18], was conducted at an inpatient psychiatric unit in which a person-centred care intervention was implemented. The intervention was associated with increased client and caregiver satisfaction, a better understanding of the client’s situation, and improved team ability to document a person-centred care plan. One of the three outcome studies included patients with schizoaffective disorder [17] although the study did not specifically target patients with a psychosis diagnosis. The other two studies [16, 18] did not report diagnostic categories. Therefore, it is unclear if the intervention outcomes can be reproduced in inpatient psychosis populations.

There are some studies indicating that persons with psychoses, despite having symptoms that might make it especially difficult for this group to actively participate in their care, also might benefit from person-centred care interventions. A systematic review and meta-analysis that focused specifically on shared treatment decision-making and patient empowerment in both in- and outpatient settings where at least half of the participants had a psychosis diagnosis showed small beneficial effects of increased shared decision-making on treatment-related empowerment [8]. Focusing specifically on treatment for persons with a psychotic illness, a randomized controlled study involving outpatients, case managers, relatives, and other significant persons in a person-centred Resource Group model showed improved social function and consumer satisfaction both at two [19] and five year [20] follow-ups.

### Aim

Considering the lack of studies specifically targeting inpatient care for persons with schizophrenia and similar psychoses, we wanted to develop and test an educational intervention for staff working in hospital services for persons with schizophrenia and similar psychoses (Person-Centred Psychosis Care, PCPC). We describe here the design and planned evaluation of the staff educational program. Specific research questions include: 1) Will the intervention have effects on empowerment and satisfaction with care in persons with schizophrenia and similar psychoses? 2) Will the intervention have effects at the ward level (duration of inpatient care, daily overall ward burden, involuntary care measures such as use of physical restraints and forced injections)? 3) What are the experiences of the care and the intervention as narrated by persons with schizophrenia and similar psychoses, by their next-of-kin, and by hospital staff?

## Methods/design

A before and after design will be used to explore quantitative outcomes of the PCPC intervention. In addition, the narrated experiences of the care and the intervention will be described.

### Participants and setting

All participants will be recruited from the Psychosis Clinic at Sahlgrenska University Hospital, Gothenburg, Sweden. This is the only clinic providing inpatient services for persons with schizophrenia and similar psychoses in Gothenburg, the second largest city in Sweden. The clinic has four inpatient wards with a total of 43 beds.

#### Patient sub-study

Fifty patients will be recruited before the intervention starts and fifty will be recruited when the intervention has been implemented at all four wards. The inclusion criteria for the persons with psychosis disorders will be: age ≥ 18 years fulfilling criteria for a clinical diagnosis of schizophrenia or other psychotic spectrum disorder in accordance with ICD-10 [21]. Individuals with co-morbidity will be included. Exclusion criteria include severe cognitive disability with inability to comprehend study goals and procedures (as determined by the patient’s psychiatrist), or lack of knowledge of the Swedish language to such a degree that an interpreter is required.

#### Staff sub-study

We will recruit a sample of staff members (*N* = 20) comprising persons from all four wards, across professions, and with varying age, gender, and length of employment. All members of staff willing to participate are eligible for inclusion and there are no exclusion criteria. However, practical circumstances such as being at work and being available for interviews on scheduled interview days might limit participation.

#### Next-of-kin sub-study

We will strive to include relatives of patients with varied backgrounds with regard to age, gender, ethnic background, education level, living situation (living together with the patient or not), and involuntary care. For relatives to be included, the patients must have agreed to us to contacting their next-of-kin. All relatives willing to participate are eligible for inclusion and there are no exclusion criteria.

### Power calculation

The primary outcome will be patient empowerment. A power calculation based on an independent samples t-test showed that a total sample size of 84 participants (42 per group in a balanced design) yields 80% power to detect a .2 difference in mean empowerment score between groups when “pre-intervention” and “post-intervention” scores are compared with a two-tailed test at a significance level of .05.

### The person-centred psychosis care intervention

The intervention is based on theoretical components of person-centred care. The person with psychotic illness is seen as a capable person who has a unique understanding of her- or himself, with unique experiences, expectations, needs, preferences, and resources [9, 22]. The success of implementation of complex care interventions depends partly on the ability to tailor the intervention to the existing care setting [23]. Therefore, we decided to use an approach built on Action Research principles [24, 25] where the patients, professionals, and researchers co-create the intervention to fit the specific care setting. The intervention was developed in two overlapping phases: an educational and experimental learning phase, and an implementation phase.

#### Educational and experimental learning phase

**Fig. 1:**
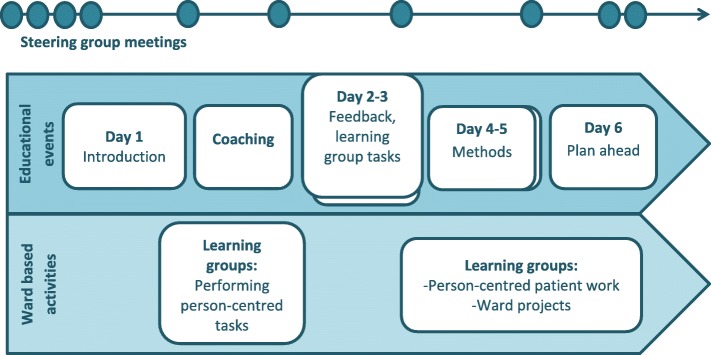
The educational and experimental learning phase process

The educational and experimental learning phase (see Fig. 1) started in December 2014 and took place over a 20 week period. Ten staff participants from each of the four hospital wards were co-creators of learning. The group (*N* = 40) included psychiatric nurses, psychiatric carers, psychiatrists, social workers, occupational therapists, and a medical secretary. There was a planned structure for the learning phase and this was modified according to the needs of the participants. The educational intervention was led by a behaviour scientist specialising in culture change in large organisations with complex dynamics.

The participants were divided into cross-disciplinary learning groups that used an experimental approach in performing person-centred tasks such as person-centred dialogue with patients and writing person-centred care plans. Experimental learning or action learning can be described as an iterative process in which a problem is formulated, a plan of how to work in order to solve the problem is formulated, the new intervention is tested, and the results are analysed, documented, and reflected upon. If the intervention solved the problem it might become standard practice, but if it did not, then the process starts over again. A description of the educational intervention follows.

The educational intervention started with an introduction to theoretical components of person-centred care. Research findings from previous person-centred care studies carried out at somatic wards at the same hospital [12, 26–28] were presented along with ways to operationalize person-centred care components: building a partnership with the patient, using dialogue, co-creating a care plan with the patient, and documenting this care plan.

The staff learning groups planned the tasks to be performed until the following education day. The first task was to have a person-centred dialouge with a patient, write a person-centred care plan, follow up the care plan, reflect upon the task and decribe in writing the work with the patient and the care plan; what worked, and what did not work. The second task was to invite two colleagues who did not participate in the educational events for a free working lunch and discuss person-centred care. The participants were supported by a coach during these tasks if needed. The coach was a psychiatric nurse and had experience of working with other person-centred care projects and therefore could give feedback regarding difficulties the participants might have when performing their person-centred tasks.

Education days two and three involved presentations of the person-centred tasks that the learning groups had performed as well as giving and receiving feedback regarding these tasks. The learning groups also discussed possible new tasks that might make the work in their wards more oriented to person-centred care. The groups were presented with a scenario in which they had successfully developed person-centred care for two years and discussed questions such as: How does the co-operation between the inpatient wards and the outpatient units work now? When you reflect over the process that led you to this point, what was crucial to get you here? Then, the groups used their discussion to formulate one activity of change that could be performed in the wards immediately, and one activity of change that could be performed within three months.

The fourth and fifth education days started with follow-up and feedback regarding the person-centred tasks the staff had performed. Then, the participants were involved in a dialouge exercise in order to formulate a crucial question regarding an activity of change that they wanted the help of the whole group to answer. An Open Space dialouge followed around the questions: How are we going to perform person-centred care with our patients? What is a person-centred care plan? How do we want the co-operation between the inpatient care and outpatient units to work? How should we relate to our patients’ will and view of the world? How can we perform person-centred care with patients who undergo involuntary treatment? How do we need to develop our existing structures and roles in order to be able to work in a person-centred way? Which challenges have we discovered when we work in a person-centred way? Which risks might there be with person-centred care? These discussions were summarized and discussed further. Thereafter, the participants worked with an implementation plan of person-centred care. The starting point for this plan was the goal; person-centred patient work. The implementation plan included several steps such as: Which important steps towards the goal have we already taken? What do we need to do within one week? What do we need to do within one month? At the end of the fifth education day, the participants had an implementation plan to work with that contained activities to be performed in the short and longer term.

The last occasion for the education intervention started with following up on the activities of change that the learning groups had performed. A discussion about what constitutes successful change followed. The learning groups then continued to work with their implementation plans. They discussed challenges, resources, and co-operation partners. They also discussed how to follow up on their short- and long-term person-centred goals. The educational intervention finished with a summary of what had been accomplished during the intervention and each learning group had a tailor-made implementation plan to follow during the implementation process.

#### Implementation phase

The implementation phase overlaps with the first phase since successful experiences are shared with colleagues on the own ward as well as the other three wards. Thus, all staff on all four wards work with and further develop the PCPC intervention together with the patients.

To further assist the development of person-centred care activities and their implementation process, a steering group and an implementation group monitor the progress, taking action when needed. Clinical supervision sessions are provided to support continued experimentation with and implementation of person-centred interventions. Various aspects of person-centred care can be discussed in connection with lectures, seminars, and small group supervision sessions.

The implementation phase lasts for approximately three years, which allows staff to develop care interventions that promote patient involvement in the care. However, since person-centred care concerns an iterative process of culture change, there is no real end to the implementation process.

### Data collection and procedures

Before any data was collected, the Regional Ethics Board in Gothenburg approved the study. Pre- and post-intervention data are collected using self-report instruments, hospital registers, and interviews.

The participants are informed about the purpose of the study. They are also informed that participation is voluntary and (for patients and next of kin) does not affect the care, that the data is anonymous, that data is kept in a safe which only the research group has access to, that it is possible to withdraw consent without explanation, that results are reported so that the identity of the participants is protected, and that results are to be reported at conferences and in scientific journals.

#### Patient data

Shortly before planned discharge, a nurse or a psychologist included in the research group asks persons with psychosis disorders to complete research questionnaires related to outcomes and possible confounding variables (specified below). Some open-ended questions are also asked.

Since the data collection is carried out during inpatient care, all the usual management and safety mechanisms are in place. Should complications occur, these are reported at the usual clinical rounds.

The primary outcome measure is self-reported empowerment. The Empowerment Scale [29, 30] is validated and used internationally in studies involving persons with severe mental ill-health. There are 28 questions; responses are given on a Likert type scale ranging from 1 (agree totally) to 4 (disagree totally), and a higher score indicates a higher level of empowerment.

The secondary outcome measure is consumer satisfaction measured with the UKU-ConSat Rating Scale [31]. This scale contains 11 items; responses are given on a Likert type scale ranging from − 3 (very bad/negative/little) to + 3 (very good/positive/much). A higher score indicates a higher degree of satisfaction with care. The scale also contains a question on overall life experience and a question concerning mental health with the answer format 0 (worst imaginable) to 100 (best imaginable).

Possible confounders including illness burden, functional ability, and overall health are quantified for each patient at discharge. Positive and negative symptom burden are rated with the Remission sub-scale of the Positive and Negative Symptoms Scale consisting of 8 items reflecting core symptoms of schizophrenia [32]. Functional ability is determined using the GAF Scale [33] and overall health with the EQ-5D Scale [34].

Both during the pre- and post- intervention periods, experiences of care during the inpatient stay are explored in individual semi-structured interviews with a purposeful sub-sample of patients. Questions include: Please describe your experiences of the care you received. What did you perceive as helpful/problematic/difficult? Did you feel listened to? Were you included in decisions about the care?

#### Staff experiences

Staff’s experiences of the educational intervention and the implementation process are explored in focus group interviews. The interviews include questions about factors that are perceived to be valuable for the development of the new care model as well as factors that are perceived to be barriers.

#### Next-of-kin experiences

The next-of-kin are invited to participate in focus group interviews. Questions include: Please describe your experiences of the care your relative received. What did you perceive as helpful/problematic/difficult? Please describe your involvement in the care if you were involved. Did you feel listened to? All interviews are recorded and transcribed verbatim.

#### Ward level data

Overall ward burden is routinely measured with a standardized questionnaire in the context of quality monitoring at the Psychosis Clinic. Other ward level data including duration of inpatient care and number of days with involuntary treatment are obtained from electronic records in use at the clinic.

#### Project timeline

**Fig. 2:**
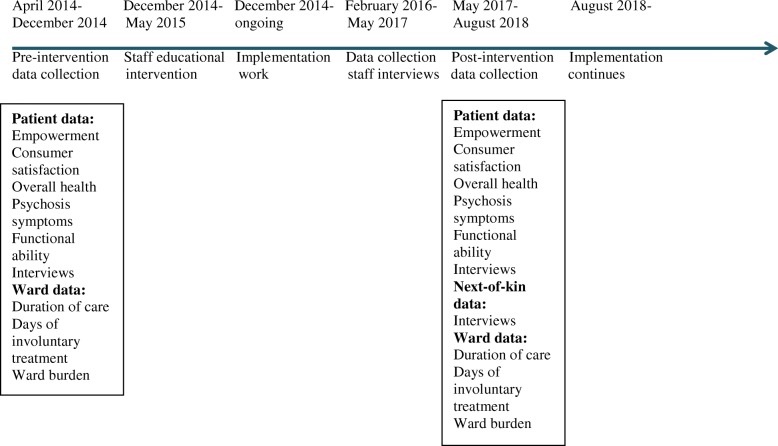
A project timeline including information about data collected at different points in time

Figure 2 shows a timeline for the project. During the pre-intervention data collection, questionnaire and interview data was collected from patients together with quantitative ward data.

After the education intervention and during the implementation process, staff interviews were performed.

During the post-intervention data collection, questionnaire and interview data is collected from patients, interviews are performed with next-of-kin, and quantitative ward data is collected.

### Data analyses

Random data entry checks will be performed by a person who is in the research group but who is not involved with data entry to promote data quality. Missing continuous data will be dealt with in line with published instructions from the researchers who developed the different instruments. The distributions of the continuous variables will be examined to determine whether parametric (independent samples ANCOVA) or non-parametric (Mann-Whitney U-test) analyses will be employed when analysing group differences. The Chi-square test will be used to analyse dichotomised data. Two-tailed tests will be used and *p*-values less than .05 will be considered statistically significant. The focus group interview data will be analysed using methods for thematic analysis [35].

## Discussion

The current study employs a simple before and after design. While this is recognized as an important limitation, randomised control trials are not always optimal for assessing complex interventions in everyday clinical practice [23]. Randomising individual patients on the same ward (PCPC vs. standard care) was not considered an alternative as there would be a risk for “contamination” of the standard care situation as the intervention will introduce a new way of thinking about care practices. A traditional cross-over design is not possible as the intervention includes a pedagogical intervention for staff that cannot be “unlearned”. Randomising two of our wards to intervention and the other two wards to standard care would not provide sufficient power for a cluster study, and would be difficult since patients and staff sometimes move between wards which would “contaminate” the standard care situation. For a true evaluation of the effects of a person-centred educational intervention for care staff, another type of study design is required. The appropriate design would be a trial randomised at group level, a cluster randomised trial [23] in which random allocation is done at the cluster level (i.e. at the level of the service providers).

The fact that there are relatively few research studies of person-centred care in inpatient psychiatric settings, and in mental health settings in general [36] might be explained by these kinds of design problems which might deter researchers from studying outcomes of person-centred care. The lack of studies might also in part be explained by implementation problems. Smith and Williams [36] conducted a review and identified an important barrier to operationalise person-centred care practices, “the reluctance of service providers to fully embrace the principles of person-centred care, particularly the challenge presented by ‘empowerment’” (p. 296). To better meet potential implementation problems, we will tailor the PCPC intervention to the existing care setting and use a participatory approach in which the patients, professionals, and researchers co-create the intervention to fit the specific care setting.

For a number of reasons including thought disorder, paranoia and reduced insight, persons with psychotic illness may be particularly sensitive when it comes to participation in research projects. The intervention will be carried out during inpatient care and thus all the usual management and safety mechanisms are in place. Should complications arise, these will be reported at the clinical rounds. Each patient has an own contact staff person, who will communicate problems if and when they arise. The ability of the staff to be flexible and understanding of the psychotic individual’s situation is paramount, as is also the case in routine clinical care of persons with severe mental illness. Members of the research team have long experience of Research and Development projects involving this patient group. Care will be taken to ensure that participants are able to understand the meaning of the study and its procedures.

If this person-centred care intervention proves beneficial, the project may provide new directions for the planning of mental health services for persons with severe mental illness. The qualitative data will help to identify specific intervention components that are experienced as helpful or as barriers, from the perspectives of the individual patient, their relatives, and psychiatric staff. This is important as the planned intervention is complex in nature.
